# Composition and metabolic potential of microbiomes associated with mesopelagic animals from Monterey Canyon

**DOI:** 10.1038/s43705-022-00195-4

**Published:** 2022-11-22

**Authors:** Corinna Breusing, Karen J. Osborn, Peter R. Girguis, Aspen T. Reese

**Affiliations:** 1 Graduate School of Oceanography, University of Rhode Island, Narragansett, RI USA; 2 Smithsonian National Museum of Natural History, Washington, DC USA; 3 Monterey Bay Aquarium Research Institute, Moss Landing, CA USA; 4 Department of Organismic and Evolutionary Biology, Harvard University, Cambridge, MA USA; 5 Division of Biological Sciences, University of California San Diego, San Diego, CA USA; 6 Center for Microbiome Innovation, University of California San Diego, San Diego, CA USA

**Keywords:** Microbiome, Microbial ecology

## Abstract

There is growing recognition that microbiomes play substantial roles in animal eco-physiology and evolution. To date, microbiome research has largely focused on terrestrial animals, with far fewer studies on aquatic organisms, especially pelagic marine species. Pelagic animals are critical for nutrient cycling, yet are also subject to nutrient limitation and might thus rely strongly on microbiome digestive functions to meet their nutritional requirements. To better understand the composition and metabolic potential of midwater host-associated microbiomes, we applied amplicon and shotgun metagenomic sequencing to eleven mesopelagic animal species. Our analyses reveal that mesopelagic animal microbiomes are typically composed of bacterial taxa from the phyla Proteobacteria, Firmicutes, Bacteroidota and, in some cases, Campylobacterota. Overall, compositional and functional microbiome variation appeared to be primarily governed by host taxon and depth and, to a lesser extent, trophic level and diel vertical migratory behavior, though the impact of host specificity seemed to differ between migrating and non-migrating species. Vertical migrators generally showed lower intra-specific microbiome diversity (i.e., higher host specificity) than their non-migrating counterparts. These patterns were not linked to host phylogeny but may reflect differences in feeding behaviors, microbial transmission mode, environmental adaptations and other ecological traits among groups. The results presented here further our understanding of the factors shaping mesopelagic animal microbiomes and also provide some novel, genetically informed insights into their diets.

## Introduction

An overwhelming majority of animals live in association with microbial organisms (bacteria, archaea, fungi, protists) that range from beneficial symbionts, essential for host nutrition, immunity, and development, to pathogenic agents promoting disease [1–3]. Increasing insights from a variety of host-microbe relationships suggest that the functions performed by these microbiomes are not only important on an organismal level, but also on an ecosystem scale, and are likely critical for facilitating ecological resilience to environmental change [4, 5].

In the marine environment, studies of animal microbiomes have primarily focused on benthic invertebrates such as corals and sponges (e.g., [6, 7]). By contrast, less is known about the composition, structure, and variability of microbes associated with the vast majority of pelagic animals, despite the significance of these organisms in global food webs and biogeochemical cycles [8]. To date, analyses of microbiota associated with whales, fishes and planktonic crustaceans have shown that microbiomes of some species can be highly host-specific and distinct from microbial consortia in surrounding seawater [9–12]. For example, herbivorous reef fishes are known to harbor species-specific resident symbionts that are critical for prey digestion [11], while North Atlantic copepods affiliate with specialized bacterial communities that are predicted to mediate key ecological processes, such as iron and phosphorus regeneration [9]. However, host specificity is not universal and other factors such as diet, habitat and seasonal changes typically also play a role in shaping microbiome composition [10, 13–15].

The mesopelagic or midwater zone (200–1000 m) represents one of the most understudied, though ecologically significant, areas of the ocean [16] and provides a unique opportunity to study how phylogenetic inertia, ecological pressures, behavioral adaptations, and biogeochemical processes shape host-associated microbiomes. For example, mesopelagic fishes are critical components of the oceanic food web, comprising approximately 10 billion tons of biomass that feed on zooplankton, and in turn provide a food source for marine mammals, birds and commercially harvested fish species such as tuna and swordfish [17, 18]. Mesopelagic organisms also constitute important links between surface and deep waters. It is estimated that about 90% of surface-derived organic matter is respired within the mesopelagic zone and consequently recycled into nutrients for photosynthetic primary production in the upper ocean [19]. Furthermore, mesopelagic animals frequently perform diel vertical migrations (DVM) to search for food and avoid predators [20], thereby promoting export of organic carbon to the deep sea [21–23]. This daily mass movement may have significant impacts on the composition of host-associated microbiota by promoting turnover of microbial communities as an adaptation to differential oxygen exposure between the oxygen-limited mesopelagic zone [24] and oxygen-rich surface waters, though this has not been formally tested. Based on evidence from metagenomic and 16S rRNA studies, DVM appears to be an insignificant driver of microbiome composition in mesopelagic zooplankton [9], but it is likely relevant for shaping the gut microbial communities of different midwater fish species, in addition to other variables such as host identity [25].

Diet can be expected to be another important factor influencing the composition of mesopelagic host-associated microbiomes. The food available at mesopelagic depths is derived principally from the photic zone, and transits passively or actively 10s–100s of meters through the water column and the gambit of hungry animals found there. Consequently, the dietary material available to mesopelagic organisms, particularly detritivores, is generally low in quality and abundance. Mesopelagic animals might thus host diverse gut microbiomes whose functional capacities could assist them in extracting nutrition from their diets. Compared to other oceanic zones, water in the mesopelagic also tends to harbor a taxonomically and functionally more diverse environmental microbial community [26], which could increase the complexity and variation of host-associated microbiomes. However, the composition of microbiota harbored by mesopelagic animals is currently largely unknown, as are the environmental factors that influence their structure and diversity.

In light of the key role that pelagic organisms play in marine ecology and biogeochemistry, we sought to better understand the patterns and processes affecting microbiome diversity and functional potential among mesopelagic animals. To that end, we employed 16S rRNA amplicon and shotgun metagenomic sequencing to samples from eleven midwater fish and invertebrate species from Monterey Canyon (Northeast Pacific Ocean). We subsequently tested for effects of trophic level, DVM, depth and host species on microbiome composition and assessed microbiome functional potential by linking microbial taxonomy and gene content to metabolic capacity.

## Materials and methods

### Sample collection and sequencing

Thirty-eight specimens of three mesopelagic fish and eight invertebrate species were collected with trawls or remotely operated vehicles (ROVs) from 239–1800 m depth during an R/V *Western Flyer* cruise (Monterey Bay Aquarium Research Institute, Moss Landing, CA) to the Monterey Canyon (36°41.94’ N, 122°2.96’ W) in June 2018 (Table 1). Sample numbers were limited by the availability of individuals for each species that could be captured through net- or ROV-based methods. On board the ship, specimens were visually identified to genus level, and classified as diel vertical migrators or non-migrators based on previous observations (Table 1) [18, 27–36]. Specimens were stored whole in RNALater™ (Thermo Fisher Scientific, Waltham, MA) except for *Tomopteris*, *Stenobrachius*, and *Vampyroteuthis*. For these genera, gut tissues and stomach contents were dissected on board ship and stored in RNALater™ as specimens were too large to be preserved whole and the remaining tissues were committed to other projects. Total DNA from *Acanthamunnopsis*, *Poeobius*, *Eusergestes*, *Tomopteris*, *Vampyroteuthis*, and *Vitreosalpa* was extracted with the Qiagen RNeasy PowerSoil DNA Elution Kit (Qiagen, Hilden, Germany), while total DNA from *Cyclothone*, *Euphausia*, *Munneurycope* and *Stenobrachius* was extracted with the Qiagen PowerSoil Kit (Qiagen, Hilden, Germany) following the manufacturer’s instructions. We made an effort to keep extraction methods as consistent as possible, though the two different protocols were necessary as different taxa, e.g., gelatinous zooplankton versus fishes, required different methods to yield high-quality DNA. Barcoded 150 bp single-end amplicon libraries of the 16S V4 rRNA region were subsequently prepared with the 515F/806R primer pairs after Caporaso et al. [37] and sequenced to an average of 72,358 reads per sample on a HiSeq 2500 instrument at the Bauer Core Facility at Harvard University. In addition, shotgun 2 × 150 bp paired-end, dual-indexed metagenomic libraries were constructed with the Nextera XT DNA Library Preparation kit in ¼ reactions (Illumina, Inc., San Diego, CA), normalized, and sequenced on a NextSeq 500/550 platform to an average of 8,223,690 total reads per sample. Metagenomic libraries were not prepared for *Vitreosalpa* samples due to insufficient DNA quality. Finally, host species identities for representative samples were verified through molecular barcoding of the cytochrome-c-oxidase subunit I (*COI*) gene with degenerate primers commonly applied for fish and invertebrate taxa [38–40].

**Table 1:** Sampling information for mesopelagic animal species in the Northeast Pacific Ocean (36°41.94’ N, 122°2.96’ W).

Species	Taxonomy	*N*	Collection depth (m)	Depth range (m)	Method	DVM	References
*Acanthamunnopsis milleri*	Arthropoda; Malacostraca; Isopoda; Munnopsidae	4	278–337	100–400	ROV	No	[32]
*Munneurycope murrayi*	Arthropoda; Malacostraca; Isopoda; Munnopsidae	3	500–900	400–1600	Trawl	No	[32]
*Cyclothone atraria*	Chordata; Actinopteri; Stomiiformes; Gonostomatidae	3	500–1800	298–4938	Trawl	No	[18, 27, 31]
*Cyclothone signata*	Chordata; Actinopteri; Stomiiformes; Gonostomatidae	1	500–1800	16–4938	Trawl	No	[18, 27]
*Stenobrachius leucopsarus*	Chordata; Actinopteri; Myctophiformes; Myctophidae	4	500–1800	31–3400	Trawl	Yes	[18, 27, 35]
*Euphausia pacifica*	Arthropoda; Malacostraca; Euphausiacea; Euphausiidae	4	500–1800	0–1000	Trawl	Yes	[28]
*Poeobius meseres*	Annelida; Polychaeta; Terebellida; Flabelligeridae	6	817	350–1300	ROV	No	[29]
*Tomopteris* sp.	Annelida; Polychaeta; Phyllodocida; Tomopteridae	2	814–909	269–1316	ROV	N.a.	[34]
*Eusergestes similis*	Arthropoda; Malacostraca; Decapoda; Sergestidae	4	239–244	0–1200	ROV	Yes	[33]
*Vampyroteuthis infernalis*	Mollusca; Cephalopoda; Vampyromorpha; Vampyroteuthidae	2	540–1700	300–3000	Trawl	No	[30]
*Vitreosalpa* sp.	Chordata; Thaliacea; Salpida; Salpidae	5	448–455	N.a.	ROV	N.a.	[36]

Samples were collected between June 21 and 25, 2018. Depth information is provided for both sampling depth and natural depth range reported in the literature. References are shown for diel vertical migration status.

*N* number of samples, *DVM* diel vertical migration, *N.a.* not available.

### Profiling of microbiome composition

We used the 16S rRNA amplicon libraries to compare microbiome composition within and among host species. Raw reads were adaptor-clipped with Trimmomatic [41] and then denoised into amplicon sequence variants (ASVs) following the Usearch-Unoise3 pipeline [42], applying a maximum error rate of 0.001, a minimum sequence length of 150 bp and a minimum base quality of 20 for filtering. The resulting ASV abundance table was imported into Qiime2 (https://qiime2.org) for taxonomic classification and phylogenetic analysis. To taxonomically assign each ASV, we used both comparisons against full length 16S rRNA sequences and a Naïve Bayes classifier trained against 150 bp fragments of the 16S V4 rRNA region that were extracted from the SILVA 138 99% reference database [43–45]. Poorly annotated chloroplast and mitochondrial sequences were further resolved through BlastN [46] searches against the NCBI non-redundant database. Taxonomic information was then used to group ASVs into microbiome components and to filter out potential contaminants, diet items, and sequence artifacts. In addition, we removed singletons and rare ASVs with less than 10 total reads to reduce bias in downstream analyses. Altogether, microbiome-associated sequences represented 39.77–97.53% (mean: 80.24%) of the total libraries. For assessing phylogenetic relationships among ASVs, we used SATé-enabled phylogenetic placement (SEPP) [47] to insert each ASV sequence into a reference tree based on a preformatted SILVA 128 99% SEPP database. Fractional abundance plots were created with the PhyloSeq package in R [48, 49], excluding samples with less than 1000 reads.

As a complement to the 16S rRNA analyses, we used Shogun [50] with the Utree method [51] on the adaptor-clipped, decontaminated shotgun metagenomic data to determine the composition of microbial taxa for each sample. However, since on average only 7.46% of metagenomic sequences could be matched to microbial taxa through this approach and ordination analyses indicated clustering of samples by number of recovered prokaryotic/viral reads (Supplementary Fig. S1), we relied primarily on the 16S rRNA amplicon data for further investigations of microbiome taxonomic composition. For all functional analyses, we nevertheless used the metagenomic data given that they better represent metabolic potential via actual gene annotations (in contrast to 16S rRNA amplicons) [52].

### Identification of diet items

We performed targeted literature searches to determine the trophic level of each host taxon and create broader diet categories for downstream analyses [53–61]. In support of these investigations, we used 16S/18S rRNA sequence information from both amplicon and metagenomic sequencing to identify potential diet items for each species. Small subunit rRNA sequences in the metagenomic datasets were assembled and annotated with PhyloFlash [62], while diet-related amplicons were analyzed as described above.

### Assessment of microbiome functional potential

We used shotgun metagenomic sequencing to assess metabolic potential from gene-level functional predictions obtained through both read- and assembly-based annotations. Raw Illumina reads were adaptor- and quality-trimmed with Trimmomatic and then mapped against the PhiX and human genomes to remove common sequence contaminants. Filtered reads were subsequently matched against the KEGG orthology database via the Shogun-Utree pipeline and co-assembled into genus-level metagenomes with MetaSPAdes [63] using kmers from 21 to 121 in 10 step increments. The prokaryotic, eukaryotic and organellar fractions of each metagenome co-assembly were separated with Tiara [64] for contigs ≥1000 bp. Eukaryotic and organellar genes were identified via MetaEuk [65] with the following settings: -s 7.5 --use-all-table-starts 1 --metaeuk-eval 0.0001 -e 100 --min-length 40. The UniRef90 collection was used as a reference database for functional annotation [66]. As the achieved sequencing depth did not allow binning of metagenome assembled genomes we analyzed the combined prokaryotic fraction for each co-assembly. Genes were predicted with Prodigal [67] in anonymous mode and functionally annotated by blasting protein sequences against the UniRef90 and RefSeq databases with an e-value threshold of 1e^–10^. The taxonomic origin of each gene in the prokaryotic fraction was assessed with the Taxize package [68] in R to filter out any remaining eukaryotic contigs that could not be classified via Tiara.

To evaluate the functional potential of each animal microbiome based on 16S rRNA abundance data, we used FaproTax [69] with an annotation database updated to the SILVA 138 taxonomy and extended by unrepresented microbial taxa via literature searches. Count data were total sum scaled before collapsing of ASVs into functional categories. Overlapping records for human-, mammal- and plant-associated categories were excluded from the final output table, resulting in assignment of 52.78% of all ASVs to 51 (non-miscellaneous) metabolic categories. Proportional abundance heatmaps for functional predictions from both 16S rRNA and metagenomic data were plotted with the ComplexHeatmap package in R [70] based on Euclidean distances.

### Associations between microbiome composition, functional potential and ecological factors

We applied principal coordinate analyses based on Bray–Curtis dissimilarities and weighted UniFrac distances in R for exploratory investigations of microbiome composition in relation to host taxon, DVM behavior, median collection depth and diet category. Count data were converted to proportions prior to distance transformation [71, 72]. To statistically determine the influence of each factor on microbiome composition and functional potential we performed unifactorial PERMANOVAs with the Vegan package in R [73] using both the 16S rRNA amplicon and shotgun metagenomic data. Differential abundance of ASVs between diel vertical migrators and non-migrators was tested via zero-inflated log-normal models implemented in MetagenomeSeq [74] based on cumulative sum scaled count data. *P* values were corrected using the false discovery rate procedure at an alpha value of 0.1. Differences in aerobic and anaerobic metabolism between both groups were determined through Wilcoxon rank tests. Correspondence between microbiome composition inferred through 16S rRNA and metagenomic sequencing was assessed through Mantel tests based on Spearman rank correlations, while differences in representation of select taxonomic groups between both methods were evaluated with paired *t*-tests. All data were inspected for normal distribution via Shapiro–Wilk tests before statistical analyses in R. In the case of deviations from normality, a non-parametric equivalent was used.

## Results

### Microbiome composition of mesopelagic animals

**Fig. 1:**
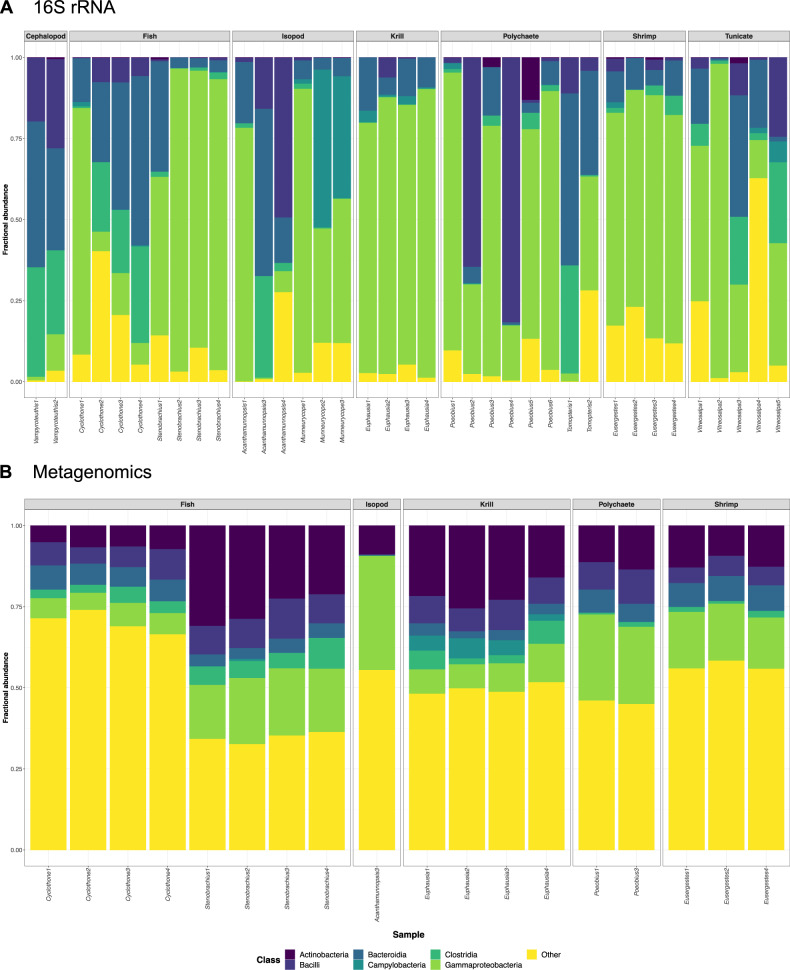
Taxonomic composition of mesopelagic host-associated microbiomes. Fractional abundances of microbial classes in the microbiomes of mesopelagic animals based on **A** 16S rRNA amplicon data and **B** metagenomic data. Gammaproteobacteria, Bacteroidia, Bacilli and Clostridia were the most prevalent bacterial classes across microbiomes based on 16S rRNA gene profiling (see also Table 2). These classes were also recovered through shotgun metagenomic sequencing, although with lower abundance. By contrast, a markedly higher proportion of Actinobacteria was revealed in each sample through this method (paired *t*-test: *t* = –8.2128, *p* < 2.548e^–07^).

16S V4 rRNA amplicon sequencing resulted in 1222 filtered microbiome-associated ASVs from 32 phyla that included at least 55 classes, 127 orders, and 226 families of microbial taxa (Supplementary Table S1). About 53.68% of these ASVs could be assigned to genus level. Despite notable variation in microbial composition among host species and individuals, the most represented bacterial classes across host-associated microbiomes were Gammaproteobacteria (mean per species: 6.18–82.96%), Bacteroidia (mean per species: 5.05–52.21%), Bacilli (mean per species: 0.43–24.98%) and Clostridia (mean per species: 0.10–29.84%) (Fig. 1 and Table 2).

**Table 2:** Most abundant family-level microbial taxa (>10% in at least one sample) in the microbiomes of mesopelagic invertebrate and fish hosts.

Host species	Microbial family	Min. [%]	Max. [%]	Mean [%]
*Acanthamunnopsis milleri*	Bacteroidota; Bacteroidia; Bacteroidales; Muribaculaceae	3.63	48.77	23.76
	Proteobacteria; Gammaproteobacteria; Burkholderiales; Comamonadaceae	0.00	57.87	19.30
	Firmicutes; Bacilli; Entomoplasmatales; Entomoplasmatales Incertae Sedis	0.00	42.26	15.23
	Firmicutes; Clostridia; Lachnospirales; Lachnospiraceae	1.41	23.96	9.11
	Proteobacteria; Alphaproteobacteria; Rhodobacterales; Rhodobacteraceae	0.00	25.44	8.48
	Proteobacteria; Gammaproteobacteria; Enterobacterales; Enterobacteriaceae	0.00	20.36	6.79
*Munneurycope murrayi*	Campylobacterota; Campylobacteria; Campylobacterales; Sulfurospirillaceae	0.00	48.56	28.65
	Proteobacteria; Gammaproteobacteria; Enterobacterales; Vibrionaceae	0.49	33.97	13.05
	Proteobacteria; Gammaproteobacteria; Pseudomonadales; Pseudomonadales MBAE14	0.00	27.53	9.19
	Proteobacteria; Gammaproteobacteria; Enterobacterales; Pseudoalteromonadaceae	0.34	19.73	7.72
	Proteobacteria; Gammaproteobacteria; Enterobacterales; Shewanellaceae	0.17	22.14	7.62
	Proteobacteria; Gammaproteobacteria; Enterobacterales; Colwelliaceae	1.69	16.50	6.84
	Proteobacteria; Gammaproteobacteria; Enterobacterales; Moritellaceae	0.15	11.58	3.97
*Cyclothone atraria*	Bacteroidota; Bacteroidia; Bacteroidales; Muribaculaceae	0.15	35.28	18.71
	Firmicutes; Clostridia; Lachnospirales; Lachnospiraceae	0.41	18.80	11.46
	Myzozoa; Conoidasida; Eucoccidiorida; Eimeriidae	0.00	36.81	15.81
	Proteobacteria; Gammaproteobacteria; Enterobacterales; Vibrionaceae	0.23	36.01	12.16
	Proteobacteria; Gammaproteobacteria; Pseudomonadales; Moraxellaceae	0.21	18.85	6.51
	Proteobacteria; Gammaproteobacteria; Enterobacterales; Pseudoalteromonadaceae	0.00	12.14	4.05
*Cyclothone signata*	Bacteroidota; Bacteroidia; Bacteroidales; Muribaculaceae	n.a.	n.a.	48.77
	Firmicutes; Clostridia; Lachnospirales; Lachnospiraceae	n.a.	n.a.	24.98
*Stenobrachius leucopsarus*	Proteobacteria; Gammaproteobacteria; Enterobacterales; Vibrionaceae	26.35	90.16	71.66
	Bacteroidota; Bacteroidia; Flavobacteriales; Flavobacteriaceae	0.83	31.10	8.96
	Proteobacteria; Gammaproteobacteria; Enterobacterales; Colwelliaceae	0.27	11.11	3.51
*Euphausia pacifica*	Proteobacteria; Gammaproteobacteria; Thiotrichales; Thiotrichaceae	13.10	53.17	26.43
	Proteobacteria; Gammaproteobacteria; Enterobacterales; Colwelliaceae	2.11	28.98	17.11
	Proteobacteria; Gammaproteobacteria; Enterobacterales; Vibrionaceae	3.87	19.87	11.21
	Proteobacteria; Gammaproteobacteria; Chromatiales; Sedimenticolaceae	0.87	18.18	8.36
	Bacteroidota; Bacteroidia; Flavobacteriales; Flavobacteriaceae	2.92	15.20	8.06
*Poeobius meseres*	Firmicutes; Bacilli; Mycoplasmatales; Mycoplasmataceae	0.00	81.71	24.37
	Proteobacteria; Gammaproteobacteria; Enterobacterales; Vibrionaceae	0.00	66.57	14.88
	Proteobacteria; Gammaproteobacteria; Nitrosococcales; Methylophagaceae	0.00	74.24	13.56
	Proteobacteria; Gammaproteobacteria; Burkholderiales; Comamonadaceae	0.00	44.05	9.65
	Proteobacteria; Gammaproteobacteria; Pseudomonadales; Moraxellaceae	0.00	31.71	8.95
	Proteobacteria; Gammaproteobacteria; Burkholderiales; Burkholderiaceae	0.00	32.06	5.34
	Proteobacteria; Gammaproteobacteria; Pseudomonadales; Halieaceae	0.00	11.02	3.02
	Bacteroidota; Bacteroidia; Bacteroidales; Muribaculaceae	0.00	14.96	2.63
*Tomopteris* sp.	Bacteroidota; Bacteroidia; Bacteroidales; Muribaculaceae	15.91	46.86	31.38
	Proteobacteria; Alphaproteobacteria; Rhizobiales; Beijerinckiaceae	0.00	23.77	11.89
	Proteobacteria; Gammaproteobacteria; Burkholderiales; Comamonadaceae	2.36	19.36	10.86
	Firmicutes; Clostridia; Lachnospirales; Lachnospiraceae	0.46	19.34	9.90
	Bacteroidota; Bacteroidia; Chitinophagales; Chitinophagaceae	0.00	16.23	8.11
	Proteobacteria; Gammaproteobacteria; Burkholderiales; Burkholderiaceae	0.10	13.33	6.72
	Firmicutes; Clostridia; Oscillospirales; Oscillospiraceae	0.00	11.23	5.62
*Eusergestes similis*	Proteobacteria; Gammaproteobacteria; Pseudomonadales; Spongiibacteraceae	40.22	61.87	50.05
	Proteobacteria; Gammaproteobacteria; Enterobacterales; Vibrionaceae	3.11	12.25	6.32
	Proteobacteria; Gammaproteobacteria; Enterobacterales; Colwelliaceae	0.82	10.74	4.10
*Vampyroteuthis infernalis*	Bacteroidota; Bacteroidia; Bacteroidales; Muribaculaceae	26.99	43.16	35.08
	Firmicutes; Clostridia; Lachnospirales; Lachnospiraceae	20.70	27.75	24.23
	Firmicutes; Bacilli; Mycoplasmatales; Mycoplasmataceae	8.94	14.44	11.69
*Vitreosalpa* sp.	Proteobacteria; Gammaproteobacteria; Nitrosococcales; Methylophagaceae	0.00	37.88	14.27
	Bacteroidota; Bacteroidia; Bacteroidales; Muribaculaceae	0.00	36.29	10.30
	Firmicutes; Clostridia; Lachnospirales; Lachnospiraceae	0.92	24.93	9.94
	Myzozoa; Conoidasida; Eucoccidiorida; Eimeriidae	0.00	45.86	9.18
	Proteobacteria; Gammaproteobacteria; Nitrosococcales; Nitrosococcaceae	0.00	33.14	8.01
	Proteobacteria; Gammaproteobacteria; Gammaproteobacteria JTB23; Unassigned	0.00	22.96	7.98
	Firmicutes; Bacilli; Entomoplasmatales; Entomoplasmatales Incertae Sedis	0.00	24.41	5.08
	Proteobacteria; Gammaproteobacteria; Pseudomonadales; Spongiibacteraceae	0.00	17.74	3.81
	Proteobacteria; Gammaproteobacteria; Enterobacterales; Colwelliaceae	0.00	12.85	2.95
	Verrucomicrobiota; Verrucomicrobiae; Verrucomicrobiales; Rubritaleaceae	0.00	11.67	2.91
	Bacteroidota; Bacteroidia; Chitinophagales; Unassigned	0.00	12.22	2.44

Range and mean percentage abundances are shown.

Microbial communities within both *Cyclothone* species and *Vampyroteuthis infernalis* were typically dominated by the bacteroidal family Muribaculaceae and the clostridial family Lachnospiraceae, whereas microbiomes of *Stenobrachius leucopsarus*, *Euphausia pacifica* and *Eusergestes similis* were primarily composed of gammaproteobacterial taxa from the families Vibrionaceae, Thiotrichaceae/Colwelliaceae, and Spongiibacteraceae, respectively (Table 2). Most *Cyclothone atraria* individuals further contained a high load of eimeriidan parasites (up to 36.81%) [75]. In contrast to the other host species, the microbial communities of *Munneurycope murrayi* were often abundant in campylobacterial taxa from the family Sulfurospirillaceae (Table 2). The microbiomes of *Acanthamunnopsis milleri*, *Poeobius meseres*, *Tomopteris* sp. and *Vitreosalpa* sp. showed the largest inter-individual variability, with either Muribaculaceae, Comamonadaceae, Entomoplasmatales Incertae Sedis, Mycoplasmataceae, Vibrionaceae, Beijerinckiaceae or Methylophagaceae being the most frequent taxa (Table 2). In virtually all samples, a single or a few ASVs dominated the microbial communities, with the most common ASVs having an abundance of up to 82.99% (Supplementary Table S2). This was particularly notable in individuals of *Acanthamunnopsis*, *Eusergestes*, *Munneurycope*, *Poeobius* and *Stenobrachius*, where usually 1–2 ASVs made up >50% of the microbiome (Supplementary Table S2). The same few ASVs or a single microbial family were typically most abundant within species of vertical migrators, while composition was more variable within species of non-migrators. Microbiome composition as determined via shotgun metagenomics was correlated with that obtained through amplicon sequencing for the unfiltered dataset (Mantel test: *p* = 0.001, *r* = 0.2486), though a significant association between both methods was not observed for the filtered dataset (i.e., samples with >1000 microbial reads). These differences could be due to data limitations resulting from the necessary filtering of the metagenomic dataset and the accompanied reduction of samples that were available for comparison. Overall, shotgun metagenomic analyses revealed a higher proportion of Actinobacteria in the host-associated microbiomes (Supplementary Fig. S2 and Supplementary Table S3; Paired *t*-test: *t* = –8.2128, *p* < 2.548e^–07^) and indicated the presence of viruses and methanogenic archaea that were missed by 16S rRNA amplicon sequencing (Supplementary Table S3).

### Inferring putative diet through 16S/18S rRNA sequence analysis

Host genera were classified into three broader categories based on diet items that were identified through literature searches (Supplementary Table S4): 1—primarily phytoplankton-based diet (*Euphausia*, *Vitreosalpa*), 2—primarily detritivorous diet (*Acanthamunnopsis*, *Munneurycope*, *Poeobius*, *Vampyroteuthis*), and 3—primarily zooplanktivorous diet (*Cyclothone*, *Eusergestes*, *Stenobrachius*, *Tomopteris*). For all species, except *Vampyroteuthis*, for which no diet-related sequences could be recovered, we complemented the literature information by inferring diet components through 16S/18S rRNA sequence analysis (Supplementary Table S4). Diet-related small subunit ribosomal sequences were mainly obtained from metagenomic data, because 16S rRNA amplicon sequencing resulted in a biased overrepresentation of diatom chloroplast sequences across all samples.

Independent of major diet category, most species contained diet components that were somewhat unexpected (Supplementary Table S4). These included genetic material from chordates, annelids, flatworms, nematodes, mollusks, cnidarians, scalidophorans, hemichordates, and copepods. The largest range of diet items were recovered for the two fish genera, *Cyclothone* and *Stenobrachius*, which seemed to feed predominantly on calanoid copepods but also gelatinous zooplankton such as cnidarians and ctenophores. Mostly crustacean sequences were detected in the other zooplanktivorous species (*Tomopteris* and *Eusergestes*).

### Influence of ecological factors on microbiome composition

**Fig. 2:**
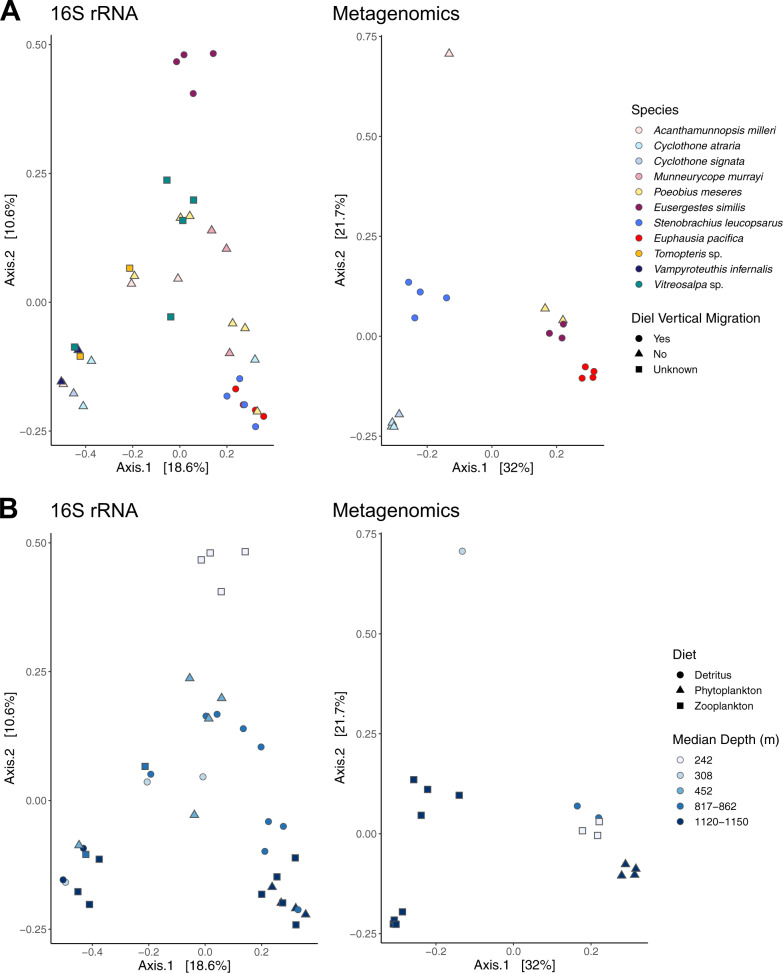
Principal coordinate analysis plots for microbiome data transformed into Bray–Curtis dissimilarities. Panels **A** and **B** show the same data colored by host (**A**) and depth (**B**), respectively, with shapes showing diel vertical migration status (**A**) and diet category (**B**). Microbial communities cluster broadly by depth and in some cases host taxon. Clustering was weaker for vertical migration status and not evident for diet, though the influence of both factors was significant in PERMANOVA (Table 3).

Principal coordinate analyses based on 16S rRNA data suggested a broad partitioning of microbiomes into (i) one shallow group (*Eusergestes*) that comprised only diel vertical migrators, (ii) two deep groups (*Cyclothone*, *Stenobrachius*, *Euphausia*, *Vampyroteuthis*) that contained half migrators and half non-migrators and species of unknown status, and (iii) one relatively diverse medium-depth group (*Acanthamunnopsis*, *Munneurycope*, *Poeobius*, *Tomopteris*, *Vitreosalpa*) that consisted mostly of non-migrators (Fig. 2). Clustering of microbiomes by host taxon was evident for *Munneurycope* (weighted UniFrac), *Eusergeste*s, *Stenobrachius*, *Euphausia* and *Vampyroteuthis* (Bray–Curtis + weighted UniFrac), while microbial communities appeared more variable between individuals of the other species (Fig. 2 and Supplementary Fig. S3). Principal coordinate plots based on shotgun metagenomic data confirmed the observed groupings by host species for *Eusergeste*s, *Stenobrachius* and *Euphausia* and by genus for *Cyclothone* (Fig. 2).

The influence of all factors (host identity, diet, migration, and depth) on microbiome composition was significant in PERMANOVAs based on Bray–Curtis dissimilarities (Table 3). Analyses based on weighted UniFrac distances also generally supported these results, except that the effect of diet was not significant (Supplementary Table S5). For most variables in analyses based on 16S rRNA amplicons, no differences in multivariate dispersion were detected, suggesting that the observed patterns were typically not biased by variance in spread among groups. The largest effect size was observed for host species and depth, which accounted for ~50% or ~91% and ~20% or ~48% of the variation in the corresponding PERMANOVAs, whereas diet and migratory behavior accounted for ~9% and ~12% or ~37% and ~15% of the variation, respectively (Table 3 and Supplementary Table S5). Overall, diet appeared to have the weakest effect on microbiome composition, as also evidenced by minimal clustering of samples by this factor in the corresponding PCoAs (Fig. 2).

**Table 3:** Results for PERMANOVAs applied to Bray–Curtis transformed microbiome data based on **a** 16S rRNA amplicons and **b** shotgun metagenomics.

Source of variation	df	SS	*R*^2^	*p*	*p*_Dispersion_
a. 16S rRNA amplicons					
Host	10	7.4976	0.49404	**0.001**	0.052
Residuals	26	7.6784	0.50596		
Diet	2	1.3922	0.09174	**0.007**	0.243
Residuals	34	13.7838	0.90826		
Migration	2	1.8344	0.12088	**0.002**	0.253
Residuals	34	13.3416	0.87912		
Depth	4	3.2905	0.21683	**0.001**	**0.001**
Residuals	32	11.8855	0.78317		
b. Metagenomics					
Host	6	3.2255	0.90916	**0.001**	**0.002**
Residuals	11	0.3223	0.09084		
Diet	2	1.3185	0.37164	**0.001**	0.101
Residuals	15	2.2292	0.62836		
Migration	1	0.5324	0.15008	**0.007**	0.869
Residuals	16	3.0153	0.84992		
Depth	3	1.6944	0.47760	**0.001**	**0.001**
Residuals	14	1.8533	0.52240		

*P* values for permutational MANOVA and permutation tests for homogeneity of multivariate dispersions are shown. Significant tests (*p* ≤ 0.05) are indicated in bold.

*df* degrees of freedom, *SS* sum of squares, *R*^*2*^coefficient of determination (proportional explained variation), *p* *p* value.

### Differences in microbial abundance related to host vertical migration

**Fig. 3:**
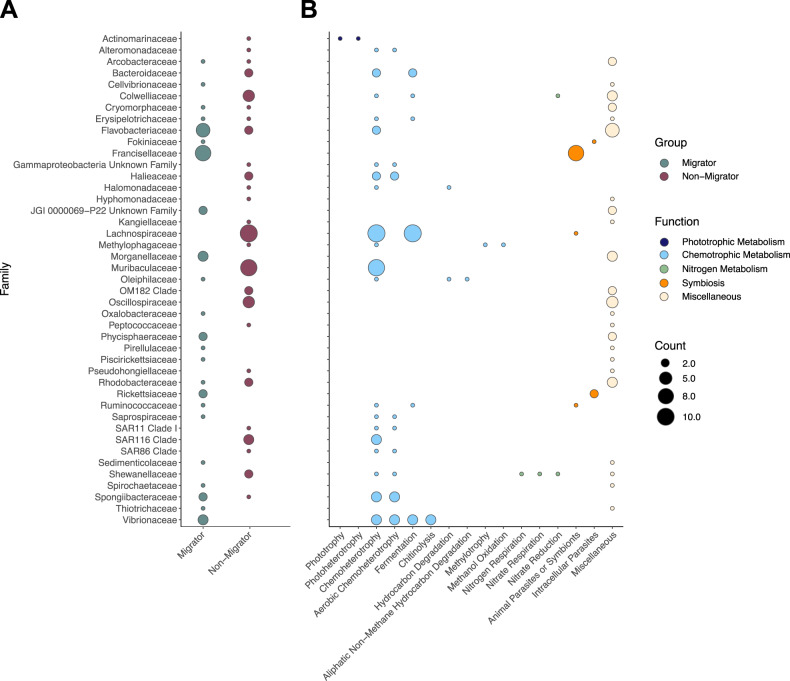
Differences in abundance of microbial taxa based on host vertical migration status. Overview of differentially abundant ASVs grouped by microbial family between diel vertical migrators and non-migrators (**A**) and their respective functional characteristics as inferred by FaproTax (**B**). A total of 100 ASVs showed significant differences in abundance based on a zero-inflated log-normal model at an alpha value < 0.1, with 57 and 43 being overrepresented in non-migrators and migrators, respectively. Dot sizes are scaled to the number of ASVs in each family (**A**) or the number of ASVs with particular metabolic functions (**B**).

A total of 100 ASVs from 43 microbial families showed significant differences in abundance between diel vertical migrators and non-migrators based on a zero-inflated log-normal model (Supplementary Table S6 and Fig. 3A). Vertical migrators were typically enriched in certain ASVs from the families Francisellaceae, Flavobacteriaceae, Morganellaceae and Vibrionaceae. ASVs from these families were predicted to be associated with symbiosis or pathogenicity (Francisellaceae), chemotrophic metabolism such as chitinolysis (Vibrionaceae) and other miscellaneous functions (Supplementary Table S6 and Fig. 3B). By contrast, non-migrators contained larger abundances of ASVs belonging to the families Lachnospiraceae, Muribaculaceae, Colwelliaceae and Oscillospiraceae (Supplementary Table S6 and Fig. 3A), which are thought to be characterized by chemoheterotrophic metabolism including fermentation and a diversity of poorly constrained functions (Fig. 3B). The strongest log-fold changes (< or > 3) were observed for 15 ASVs from the families Francisellaceae (logFC: 3.11–4.99), Vibrionaceae (logFC: 3.23), Flavobacteriaceae (logFC: 3.27–5.92), Arcobacteraceae (logFC: 3.36), Morganellaceae (logFC: 3.99–5.36), Ruminococcaceae (logFC: 4.22), Spirochaetaceae (logFC: 5.06) and Thiotrichaceae (logFC: 6.70), which were frequent in diel vertical migrators (especially *Euphausia* and *Stenobrachius*). In addition, one Bacteroidaceae ASV (logFC: –4.55) was particularly abundant in certain non-migrators (*Acanthamunnopsis*, *Cyclothone*).

### Metabolic potential of mesopelagic host-associated microbiomes

**Fig. 4:**
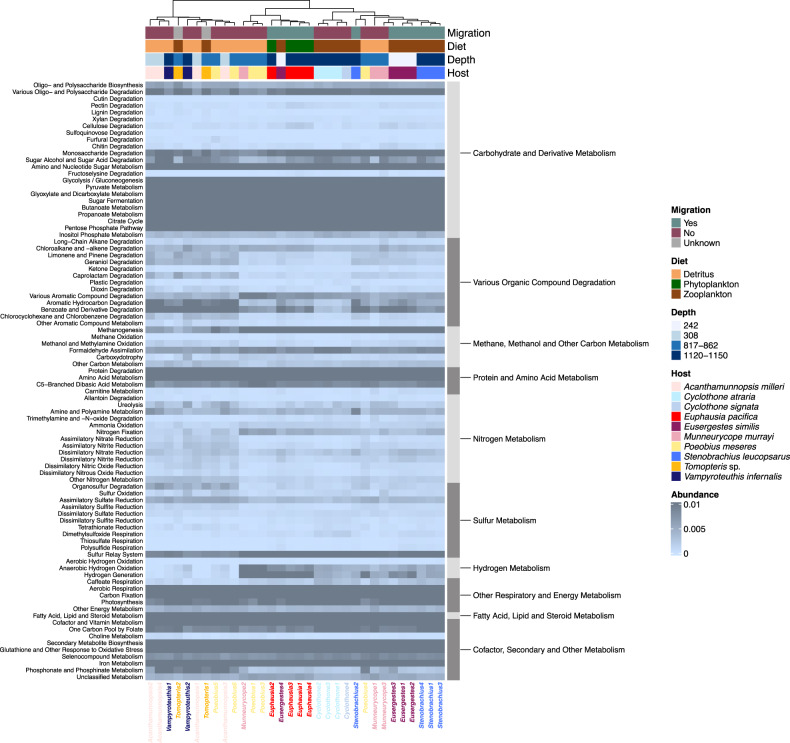
Proportional abundance heatmap based on Euclidean distance for metabolic functions predicted from gene-level shotgun metagenomic data. Samples were clustered with the complete linkage method, where similarity between clusters is determined by the distance between the most dissimilar members of the respective clusters. Functional potential shows only weak associations with diel vertical migration status and diet, but is partly linked to host taxon and depth as indicated by hierarchical clustering between samples and PERMANOVAs (Table 4). Note that genes might belong to multiple metabolic categories.

Gene-level metagenomic analyses using both read- and assembly-based annotations indicated that all microbiomes were abundant in a diversity of enzymes related to heterotrophic metabolism (e.g., peptidases, lipases and/or glucosidases) and major biochemical pathways, such as glycolysis, citric acid cycle, pentose phosphate pathway and aerobic respiratory chain (Fig. 4 and Supplementary Tables S7 and S8). In addition, genes that were linked to more specialized metabolic functions were recovered in all microbiomes, with aromatic compound and chlorinated hydrocarbon degradation, methanogenesis, formaldehyde assimilation and photosynthesis being among the most dominant metabolisms (Fig. 4 and Supplementary Tables S7 and S8). Some of the observed microbiome functions were directly related to the trophic ecology of the host organism. For example, in line with the seasonal phytoplankton-dominated diet of *Euphausia*, the microbiome of this species contained genes for endoglucanases, cellobiosidases, polysaccharide lyases and MR_MLE domain-containing proteins, which are involved in the degradation of algal cell wall compounds (i.e., cellulose, pectin and lignin-derived molecules [76]). All microbiomes showed potential for fermentative and other anaerobic metabolism. The microbial communities of most detritus-feeders encoded multiple genes for dissimilatory reduction of nitrogen and sulfur compounds, while the microbiomes of zooplanktivores and herbivores were abundant in genes for anaerobic hydrogen oxidation and generation. The proportion of anaerobic metabolism within host-associated microbiomes did not significantly differ between vertical migrators and non-migrators (Wilcoxon rank test: *W* = 150, *p* = 0.07879). Other than digestive and respiratory functions, all microbiomes showed biosynthetic potential relevant for host nutrition and host-microbe interactions, such as synthesis of amino acids, secondary metabolites, vitamins and other enzyme cofactors (Fig. 4 and Supplementary Tables S7 and S8). The influence of all tested explanatory factors (host species, depth, diet, and DVM) on the representation of metabolic potential was significant in unifactorial PERMANOVAs, with host species and depth being the strongest explanatory variables (Table 4). Despite notable inter-individual variability, clustering of microbiome functional potential by host species was evident for *Eusergestes*, *Stenobrachius*, *Euphausia*, *Cyclothone* and—to a lower extent—*Munneurycope*, in accordance with patterns observed in principal coordinate analyses based on taxonomic composition (Fig. 4). Functional predictions from 16S rRNA data supported the overall inferences from metagenomic data, suggesting a predominance of aerobic chemoheterotrophic and fermentative metabolism in all microbiomes. However, compared to patterns obtained from shotgun metagenomics, potential for chitinolysis was notably more prevalent, whereas other categories such as methanogenesis were not represented (Supplementary Fig. S4 and Supplementary Table S9).

**Table 4:** PERMANOVAs assessing abundance of metabolic categories in relation to host species, depth, diet and diel vertical migration based on **a** 16S rRNA amplicon data and **b** shotgun metagenomic data.

Source of variation	df	SS	*R*^2^	*p*	*p*_Dispersion_
16S rRNA data					
Host	10	2.5220	0.4591	**0.001**	0.157
Residuals	26	2.9712	0.5409		
Diet	2	0.5843	0.1064	**0.027**	0.114
Residuals	34	4.9089	0.8936		
Migration	2	0.5892	0.1073	**0.025**	0.300
Residuals	34	4.9041	0.8928		
Depth	4	1.0597	0.1929	**0.012**	**0.007**
Residuals	32	4.4335	0.8071		
Metagenomic data					
Host	9	0.1357	0.6726	**0.001**	**0.008**
Residuals	22	0.0661	0.3274		
Diet	2	0.0497	0.2463	**0.004**	**0.030**
Residuals	29	0.1521	0.7537		
Migration	2	0.0344	0.1706	**0.024**	**0.001**
Residuals	29	0.1674	0.8295		
Depth	3	0.0620	0.3073	**0.003**	**0.017**
Residuals	28	0.1398	0.6927		

Data were transformed into Bray–Curtis dissimilarities for analysis. P values for permutational MANOVA and permutation tests for homogeneity of multivariate dispersions are shown. Significant tests (*p* ≤ 0.05) are indicated in bold.

*df* degrees of freedom, *SS* sum of squares, *R*^*2*^ coefficient of determination (proportional explained variation), *p* *p* value.

## Discussion

Host-associated microbiomes are now appreciated as critical mediators of animal functioning and fitness [1], yet their composition, diversity and metabolic potential remains understudied in many marine environments, especially the pelagic realm. Here, we used 16S rRNA amplicon and metagenomic sequencing to gain some of the first insights into the taxonomic and functional diversity of microbial communities associated with a broad diversity of midwater fish and invertebrate species to begin to assess the influence of host taxon and environment on microbiome structure. Though the modest sample size (a consequence of logistical constraints) limited our ability to fully resolve the differential influence of host phylogeny, dietary preferences and migratory behavior, our analyses reveal key similarities and differences in microbiome composition and function among the diverse host taxa. The data provide a number of plausible suggestions (discussed in the paragraphs below) that we hope will provide the basis for future, more directed research efforts.

In accordance with observations in other pelagic animals (e.g., [9, 15, 77]), our analyses indicate that most host taxa investigated in this study harbor microbiomes dominated by Proteobacteria, Firmicutes and/or Bacteroidota (Fig. 1 and Table 2). This pattern contrasts with studies of free-living microbial oceanic communities, which are dominated by Proteobacteria but tend to have limited Bacteroidota and little to no Firmicutes [26, 78]. When looking at lower microbial taxonomic levels, we observed marked variability in microbiome composition between host species and individuals that did not necessarily agree with previous findings. For example, previous targeted and shotgun metagenomic analyses have shown that the microbiomes of planktonic crustaceans from the North Atlantic are usually abundant in Alphaproteobacteria, Actinobacteria and/or lineages from the bacteroidal order Flavobacteriales [9, 79], whereas our study suggests enrichment in gammaproteobacterial or campylobacterial taxa in Northeast Pacific shrimp, krill and isopod species (Table 2). Likewise, recent 16S rRNA profiling analyses imply that myctophid and gonostomatid fishes predominantly host Mycoplasmatales and Pseudomonadales [25] in contrast to Enterobacterales and Bacteroidales as observed in our work (Table 2). These discrepancies could be caused by species-specific variation in microbiome composition and/or differences in location and time of sample collection. In addition, methodological differences stemming from the use of whole animal samples instead of isolated guts might contribute to these patterns, revealing differences in external versus internal microbial communities.

While microbiomes were, in some cases, relatively distinct for particular host taxa, this specificity was unrelated to host phylogenetic divergence and instead correlated with other host traits. For example, all diel vertical migrators (*Euphausia*, *Eusergestes*, *Stenobrachius*) contained microbiomes that appeared to be host-specific, whereas many non-migrators showed comparatively strong inter-individual variation in their microbial assemblages (Fig. 2). Given the prevalence of horizontal transmission in aquatic systems [80], marine organisms typically acquire their microbiomes from the environment. Vertical transmission, when present, can increase consistency in composition between individuals, but strong selection can also result in homogenous communities acquired from the environment. It is plausible that the vertically migrating species investigated here inherit part of their microbiome and/or that only a limited suite of horizontally acquired microbial taxa can tolerate the highly variable environments their hosts traverse. Mesopelagic vertical migrators experience strong fluctuations in oxygen levels, which could select for stable pools of facultative anaerobes and/or promote shifts between predominantly aerobic and anaerobic microbial communities depending on the prevailing oxygen conditions. Our data do not fully allow disentangling these alternatives given that each sample was only obtained from a single timepoint and depth range. In addition, differences in host immune or physiological control might contribute to discrepancies in microbiome composition between individuals and taxa, making it difficult to distinguish the origin or mechanism of microbial acquisition and maintenance. However, depth appeared to have at least a partial effect on microbial community composition (Table 3) as microbiomes were typically more similar among individuals collected within comparable depth ranges (Fig. 2). Environmental microbial communities in Monterey Bay have been found to be vertically stratified [78], exposing host organisms to distinct microbial pools as they traverse across depths. While these patterns might contribute to some of the differences and similarities observed among host-associated microbiomes, the microbial profiles obtained in this study do not mirror environmental ones [78], as the dominant host-associated taxa were not abundant in seawater. This suggests that all host organisms (independent of the degree of host-microbe specificity) discriminate among free-living microbial groups and/or induce selective pressures on colonizing microbes that result in different microbial assemblages between hosts and their environment.

The larger intra-specific variability in non-migrators compared to migrators might also be linked to contrasting feeding habits among these groups, although the effect of diet could only be partially assessed given that our study focused primarily on whole-body microbiomes, rather than isolated digestive tracts (with the exception of *Tomopteris*, *Stenobrachius* and *Vampyroteuthis*). Nevertheless, most non-migratory species available here were detritus feeders (Table 1 and Supplementary Table S4), which likely encounter a relatively broad range of diet items and might thus experience frequent shifts in microbial community composition. On the other hand, the migratory species available here were zooplanktivores or omnivores characterized by more selective feeding behaviors (Table 1 and Supplementary Table S4), which might promote microbiome stability by limiting variation in diet components. These interpretations are in line with previous observations that differences in feeding habits and diet can influence microbiome structure among and within aquatic fish and invertebrate species [11, 15, 81–83].

Diets of many mesopelagic animals are undocumented and must be inferred from morphology or existent information in related taxa, which may or may not apply to the animal of interest. Several diet components that we recovered in this study were rather unexpected. For example, we found chordate sequences in the isopods *Acanthamunnopsis* and *Munneurycope*, the polychaete worm *Poeobius meseres*, and the krill species *Euphausia pacifica* despite that all these animals have primarily phytoplankton- or detritus-based diets (Supplementary Table S4). Similarly, we found evidence of phytoplankton in the zooplanktivorous taxa, *Eusergestes*, *Cyclothone*, *Stenobrachius*, and *Tomopteris*. Both of these findings can be explained in several ways. First, marine snow, consisting of microaggregates, organic exudate, sloughed cells, fecal pellets, phyto- and zooplankton [84], is ubiquitous and abundant and, in this form, likely contains remnants of all inhabitants of the midwater. Even if they are not primarily detritivores, all midwater animals are likely to ingest at least some marine snow along with their primary food items. Second, many of the dietary items returned (e.g., hemichordates, mollusks, flatworms, annelids, nematodes, and scalidophorans) could have been picked up from the midwater as larvae or parasites, entangled in marine snow, or ingested secondarily. In particular, *Tomopteris* has a proboscis ideally suited for attaching to and sucking up portions of gelatinous prey [85] and has been reported to feed on pelagic tunicates, chaetognaths, and sometimes, diatoms [86], yet we found primarily crustacean and phytoplankton sequences as their likely prey items. It may well be that crustaceans were secondarily ingested after being concentrated in the gut of a ctenophore or cnidarian. Finally, as with any identification of animal traces based on genetic sequencing, there are many issues with identification of the returned sequences, most having to do with the inclusiveness and coverage of the reference database [87]. The explanations for each of the returned dietary items is likely some combination of each of these scenarios, but despite these uncertainties, the returns suggest that we have much to learn about individual midwater species’ diets and now have new leads to follow up on.

Not surprisingly, metabolic diversity of the host-associated mesopelagic microbial assemblages mirrored their taxonomic diversity. Although all microbiomes showed potential for heterotrophic metabolism that suggests a predominantly digestive function, they appeared to vary in the utilization of predicted organic carbon sources (Fig. 4, Supplementary Fig. S4, and Supplementary Tables S7–S9). For example, the microbiomes of the omnivore *Euphausia pacifica* contained potential for the degradation of algal cell wall compounds such as cellulose and pectin, in line with the seasonally phytoplankton-dominated diet of this host taxon. Similarly, in accordance with the crustacean-rich diets of their hosts, the microbial communities associated with *Stenobrachius*, *Cyclothone* and *Eusergestes* were abundant in taxa from the family Vibrionaceae, which are principle chitin degraders in marine environments [88]. Surprisingly, though, chitinolytic potential appeared to be low compared to other metabolic functions based on gene-level metagenomic analyses (Fig. 4). This could indicate a limitation of the functional annotation database or that only select Vibrionaceae in these taxa degrade chitin. Apart from digestive potential directly linked to their preferred prey items, the carnivores and seasonal herbivores investigated here displayed a broad range of other microbiome functions, possibly as a result of opportunistic feeding on detrital matter.

Marine snow and fecal pellets, the dominant food sources for marine detritivores, can vary greatly in organic composition and biochemical properties based on origin, microbial activity on particles, as well as depth and the respective level of degradation [84, 89]. Detritus-feeding mesopelagic animals can therefore be expected to host metabolically diverse microbiomes that are able to utilize a variety of energy sources. Consistent with this hypothesis, the microbiomes of the exclusively detritus-feeding species showed the highest inter-individual variability in both taxonomic and metabolic diversity.

Independent of feeding mode, many individual animals harbored bacterial microbes capable of photosynthetic primary production despite the fact that light intensity in the mesopelagic zone is too low for photosynthesis. It is most plausible that these bacteria do not represent viable members of the animals’ microbiomes but were instead ingested together with the hosts’ food items. Analytical techniques distinguishing between active and inactive or dead cells would be necessary to confirm this inference. Our analyses also highlight discrepancies between methods in inferring microbiome composition and function. This could be due to low read depth, preferential amplification, or insufficient reference databases for the microbial organisms under study, underlining the need for further investigations.

In summary, our data reveal that host-associated microbial communities in the midwater animals studied here are most prominently influenced by host taxon and depth, followed by migratory behavior and diet. Given that depth equates to likely differences in food quality and quantity, we posit that mesopelagic host-associated microbiomes may have functional attributes that are effective in enabling the host to derive greater nutritional benefits from the available food sources—as evidenced by the diversity of digestive and biosynthetic functions recovered from the microbiomes. Our data further reveal that vertical migrators appear to harbor species-specific microbial taxa more commonly than non-migrators. There are a myriad of reasons that could explain this pattern, including differences in microbial transmission modes, adaptations to fluctuating oxygen concentrations, and immuno-physiological host control. However, these results also raise the possibility that the microbiomes of vertical migrators may reflect the expected differences in diet or environmental microbial communities that are encountered in the hosts’ shallower and deeper ranges. For example, the diversity of microbial communities associated with vertically migrating freshwater crustaceans fluctuates with the host’s daily feeding and resting cycle between shallow depths at night and deeper depths during the day [83]. By contrast, day-night shifts in microbiome composition were not observed in North Atlantic zooplankton communities [9]. Additional variables such as seasonal changes [10] that we could not account for in this study are likely relevant for shaping microbiome composition and functions in the mesopelagic. In species from Monterey Canyon, temporal variation of host-associated microbiomes can be particularly expected to occur over the year, given the strong oscillations in environmental conditions and planktonic community composition caused by seasonal upwelling events [78, 90, 91]. Future studies monitoring microbiome composition within and between mesopelagic species across seasonal and diurnal cycles will be helpful to address the importance of these factors in addition to other parameters such as host identity, diet, depth, and behavior. Altogether our data provide insights into the ecology and potential physiological capacities of mesopelagic host-associated microbiomes and set the stage for further inquiries that can elucidate the role of these microbiomes in shaping midwater ecological and biogeochemical processes.

## Supplementary information

Supplementary Material

Supplementary Tables

## Data Availability

Raw 16S rRNA amplicon and shotgun metagenomic reads are available at the National Center for Biotechnology Information under BioProject number PRJNA801405. Host mitochondrial *COI* sequences have been deposited in GenBank under accession numbers OM753075–OM753095 and OM753097–OM753099. Bioinformatic scripts for analysis can be found on GitHub (https://github.com/cbreusing/Marine_animal_microbiomes).
