# Supplementary material for: Composition and metabolic potential of microbiomes associated with mesopelagic animals from Monterey Canyon
Source: ISME Commun. 2022 Nov 22;2:117. doi: 10.1038/s43705-022-00195-4 (PMC9723714; doi:10.1038/s43705-022-00195-4)
Supplement: Supplementary file 1 — Supplementary Material [file 43705_2022_195_MOESM1_ESM.pdf]

Supplementary Material for

Composition and metabolic potential of microbiomes associated with mesopelagic animals from Monterey Canyon

Corinna Breusing, Karen J. Osborn, Peter R. Girguis, and Aspen T. Reese*

***Email:** areese@ucsd.edu

This PDF file includes:

Figure S1 to S3

Legends for Tables S1 to S9

Other supplementary materials for this manuscript include the following:

Tables S1 to S9

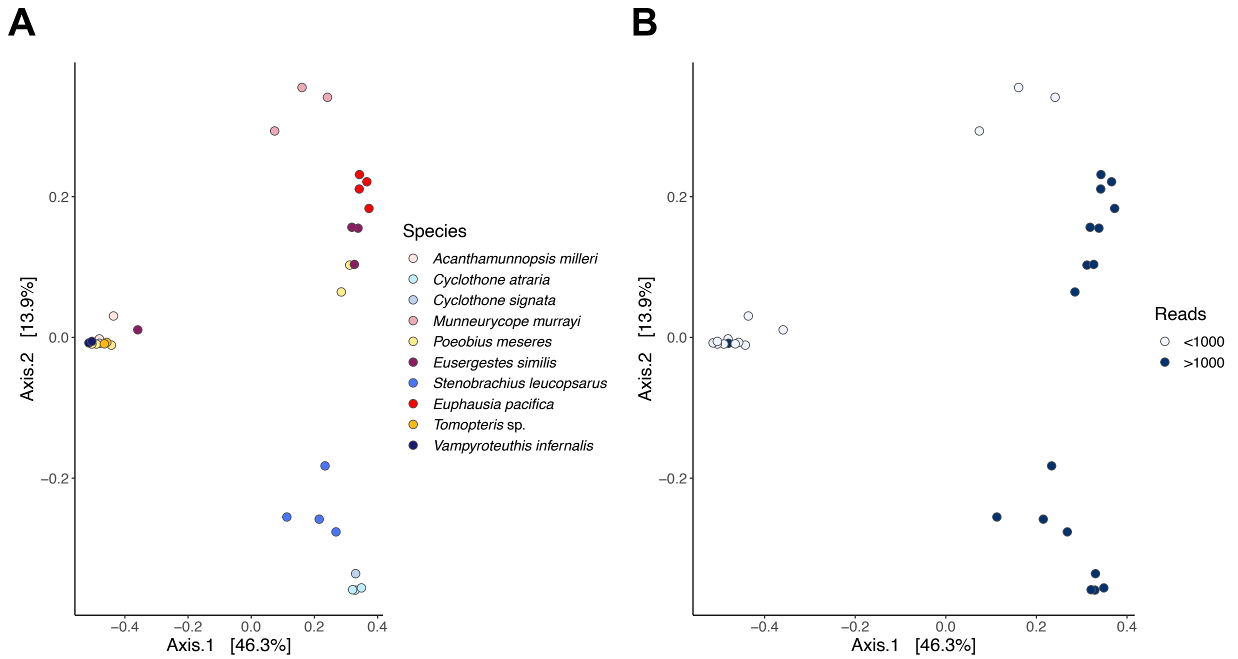

Fig. S1 Principal coordinate plots for the unfiltered microbiome dataset inferred from shotgun metagenomics. Data were normalized by library size prior to transformation into Bray-Curtis dissimilarities. In agreement with patterns in the 16S rRNA amplicon data, microbial communities cluster by host species (A), though some groupings are clearly influenced by the number of obtained prokaryotic and viral reads (B) (PERMANOVA: df = 1, SS = 3.0086, R^2 = 0.31711, $p = 0.001$, $p_{\text{Dispersion}} = 0.491$).

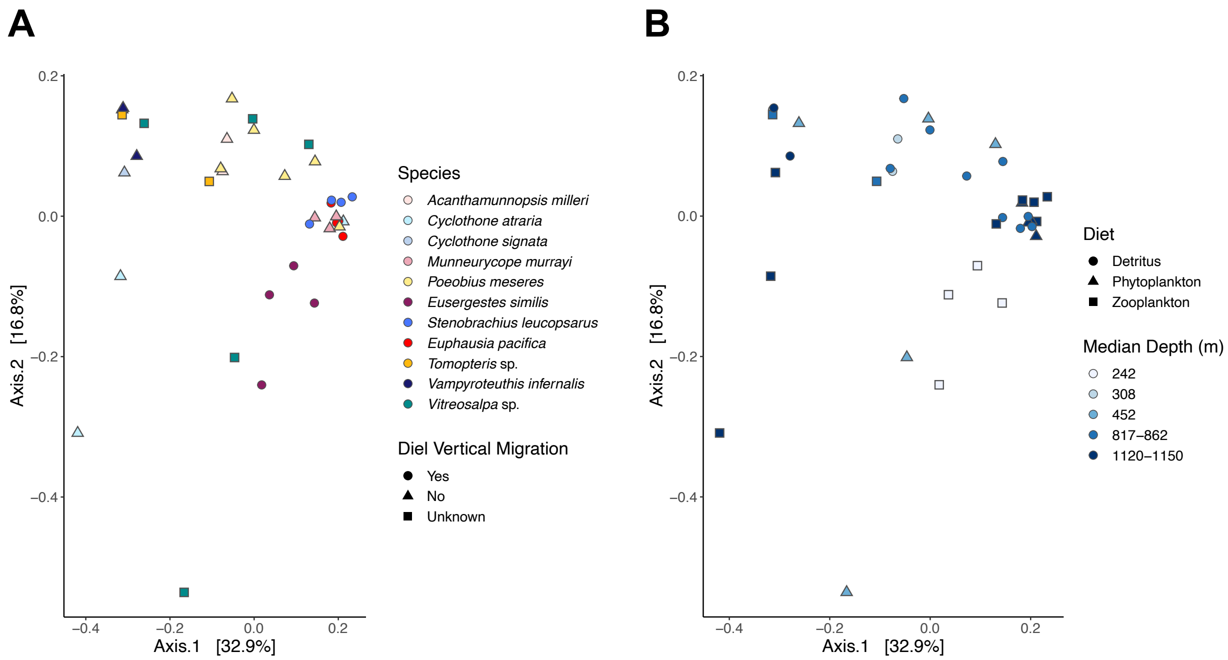

Fig. S2 Principal coordinate analysis plots for 16S rRNA microbiome data transformed into weighted UniFrac distances. Panels A and B show the same data colored by host (A) and depth (B), respectively, with shapes showing diel vertical migration status (A) and diet category (B). The effect of all factors except diet was significant in unifactorial PERMANOVAs (Table S5).

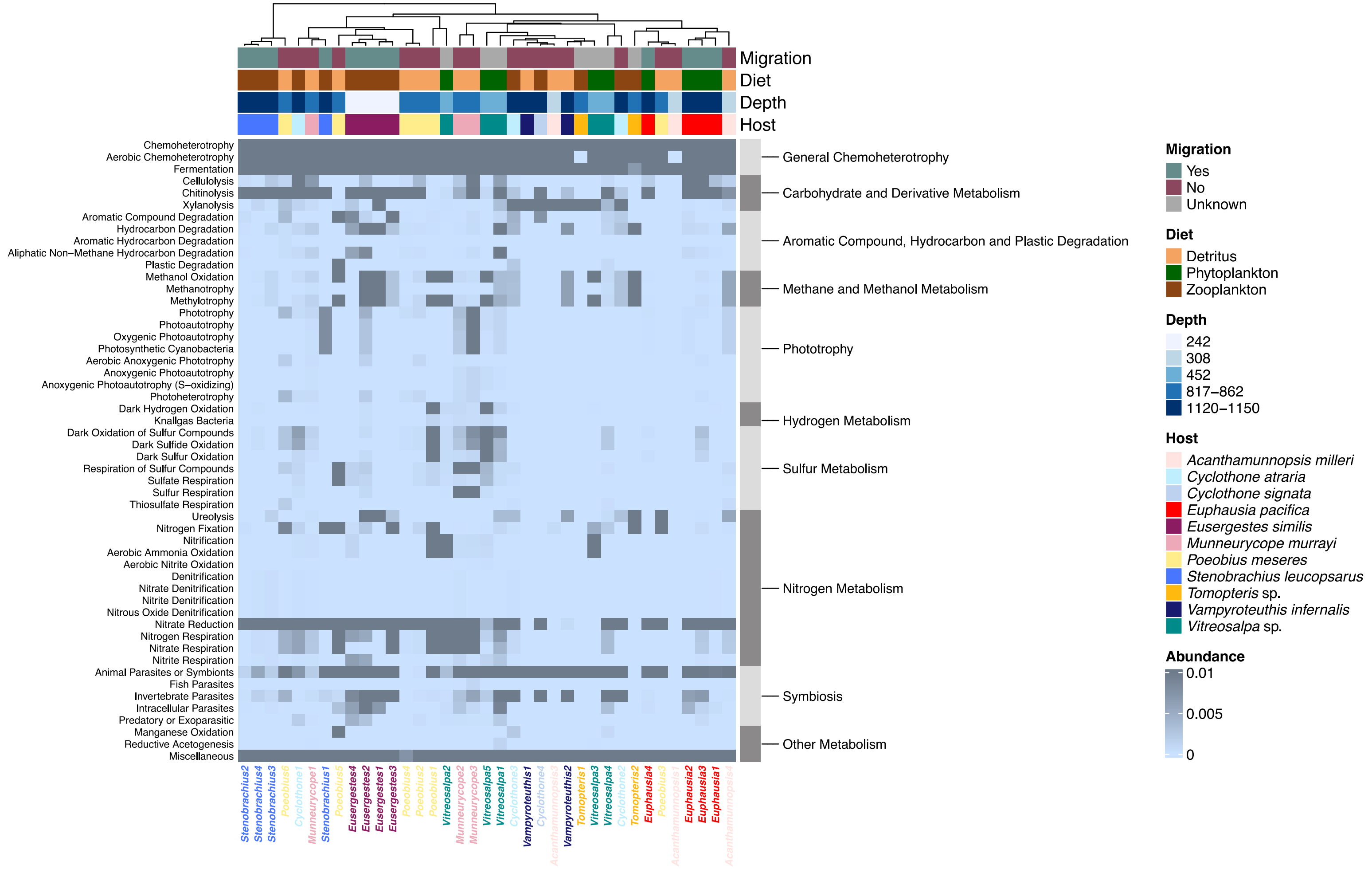

Fig. S3 Proportional abundance heatmap based on Euclidean distance for metabolic functions predicted from 16S rRNA community composition via FAPROTAX. Samples were clustered with the complete linkage method, where similarity between clusters is determined by the distance between the most dissimilar members of the respective clusters. Functional potential shows only weak associations with diel vertical migration status and diet, but is partly linked to host taxon and depth as indicated by hierarchical clustering between samples and PERMANOVAs (Table 4). Note that some functions are subgroups of broader metabolic categories and functional information for some microbial taxa is therefore redundantly represented.

Table S1 ASV abundance and taxonomic classifications for mesopelagic fish and invertebrate gut microbiomes based on filtered 16S rRNA amplicon data.

Table S2 Percentage abundances of the top 5 most frequent ASVs in each sample.

Table S3 OTU abundance and taxonomic classifications for mesopelagic fish and invertebrate gut microbiomes based on filtered shotgun metagenomic data. Only samples with > 1000 reads were included in further analyses.

Table S4 Putative diet items and categories of mesopelagic species identified through 16S rRNA amplicon and shotgun metagenomic sequencing. Probable detrital matter was inferred through cross-comparisons with known feeding strategies for each genus. Phytoplankton sequences were abundant in all samples based on 16S rRNA amplicon sequencing and might in some cases just be entrained with seawater during feeding instead of being true prey items.

Table S5 PERMANOVAs for weighted UniFrac transformed microbiome data obtained through 16S rRNA amplicon sequencing.

Table S6 ASVs with significantly different abundance between diel vertical migrators and non-migrators.

Table S7 Putative biosynthetic, digestive and respiratory functions of host-associated microbiomes based on filtered shotgun metagenomic reads.

Table S8 Putative biosynthetic, digestive and respiratory functions of host-associated microbiomes based on assembled shotgun metagenomic data.

Table S9 Binary assignment of ASVs into functional categories based on FAPROTAX. Unassigned taxa are not shown.
